# High Id1 expression, a generally negative prognostic factor, paradoxically predicts a favorable prognosis for adjuvant paclitaxel plus cisplatin therapy in surgically treated lung cancer patients

**DOI:** 10.18632/oncotarget.2595

**Published:** 2014-10-18

**Authors:** Yu-Jen Cheng, Yi-Chen Lee, Wen-Chin Chiu, Jen-Wei Tsai, Yu-Han Su, Amos C. Hung, Po-Chih Chang, Chih-Jen Huang, Chee-Yin Chai, Shyng-Shiou F. Yuan

**Affiliations:** ^1^ Division of Thoracic Surgery, Department of Surgery, and Cancer Center, E-DA Hospital, Kaohsiung, Taiwan; ^2^ Department of Postgraduate Medicine, School of Medicine for International Student, I-Shou University, Kaohsiung, Taiwan; ^3^ Department of Respiratory Therapy, College of Medicine, Kaohsiung Medical University, Kaohsiung, Taiwan; ^4^ Division of Thoracic Surgery, Department of Surgery, Kaohsiung Medical University Hospital, Kaohsiung Medical University, Kaohsiung, Taiwan; ^5^ Department of Anatomic Pathology, E-DA Hospital, Kaohsiung, Taiwan; ^6^ Translational Research Center, Department of Medical Research, and Department of Obstetrics and Gynecology, Kaohsiung Medical University Hospital, Kaohsiung Medical University, Kaohsiung, Taiwan; ^7^ Division of General Surgery, Department of Surgery, E-DA Hospital, Kaohsiung, Taiwan; ^8^ Department of Radiation Oncology, Kaohsiung Medical University Hospital, Kaohsiung Medical University, Kaohsiung, Taiwan; ^9^ Department of Pathology, Kaohsiung Medical University Hospital, Kaohsiung Medical University, Kaohsiung, Taiwan; ^10^ School of Medicine, College of Medicine, Kaohsiung Medical University, Kaohsiung, Taiwan

**Keywords:** Id1, non-small-cell lung cancer, oncogene addiction, disease-free survival, overall survival, adjuvant chemotherapy

## Abstract

Adjuvant chemotherapy is commonly given to surgically treated non-small-cell lung cancer (NSCLC) patients. However, the prerequisite for chemotherapy needs to be scrutinized in order to maximize the benefits to patients. In this study, we observed that NSCLC cells with high Id1 protein expression were vulnerable to the treatment of paclitaxel and cisplatin. In addition, paclitaxel and cisplatin caused Id1 protein degradation through ubiquitination. In the nude mice xenograft model, the tumor growth was reduced to a large degree in the Id1-overexpressing group upon treatment with paclitaxel and cisplatin. Furthermore, immunohistochemical staining for Id1 followed by Kaplan-Meier survival analysis showed that surgically treated NSCLC patients with high Id1 expression in primary tumor tissues had better disease-free and overall survivals after adjuvant paclitaxel and cisplatin chemotherapy. In summary, our current data suggest that Id1, a generally negative prognostic factor, predicts a favorable prognosis in the case of surgically treated NSCLC patients receiving the definitive adjuvant chemotherapy. The distinct role of Id1 reported in this study may arise from the phenomenon of Id1 dependence of NSCLC cells for survival, which renders the cancer cells additionally susceptive to the adjuvant chemotherapy with paclitaxel and cisplatin.

## INTRODUCTION

Lung cancer is one of the leading causes of death from cancer worldwide [1]. More than 85% of lung cancer cases belong to the non-small-cell lung cancer (NSCLC), and complete resection has been used as one of the major treatment modalities for NSCLC [1]. Surgery alone for NSCLC patients, however, shows a high recurrence rate with the 5-year recurrence ranging from 25% in stage IA to 75% in stage IIIA [2]. Notably, adjuvant chemotherapy, especially the platinum-based adjuvant therapy, advantageously reduces the recurrence and improves patient survival in surgically resected NSCLC [2-4].

Id (inhibitor of differentiation and DNA binding) proteins are helix-loop-helix proteins that form heterodimers with bHLH (basic helix-loop-helix) transcription factors to inhibit the binding of bHLH transcription factors to DNA [5]. The gene knockout or protein knockdown studies reveal that Id proteins are essential for cell proliferation and maintenance of cell viability [6-8]. For example, double knockout of Id1 and Id3 showed embryonic lethality in mice [9]. In addition to that, Id proteins act as important regulators for tumor growth, vascularization, invasiveness and metastasis [10, 11].

Id proteins consist of four subtypes, namely Id1, Id2, Id3, and Id4 [10, 12]. Among the four Id subtypes expressed in primary human malignancies, Id1 has been shown strongly correlated with various types of tumors, including lung cancer [13, 14]. For instance, our group previously reported that Id1 was abundantly expressed in human NSCLC cell lines, while the expression level of Id1 in normal lung cells was low [15]. Clinically, high Id1 protein levels were present in the tumors of NSCLC compared with normal lung tissues [16, 17]. Moreover, high Id1 expression was associated with a poor survival rate and the resistance to chemotherapy or radiotherapy, or both, in NSCLC patients [18, 19].

In this study, we investigated the role of Id1 expression in NSCLC cells treated with paclitaxel and cisplatin by *in vitro* and *in vivo* assays. Furthermore, we carried out clinical-statistical analysis to examine the association between Id1 expression and treatment outcome in surgically resected NSCLC patients followed by definitive adjuvant paclitaxel and cisplatin chemotherapy. Our current data indicated that, while overexpression of Id1 promoted NSCLC cell growth, co-treatment with paclitaxel and cisplatin led to a greater reduction of the growth of NSCLC cells overexpressing Id1 compared with that of control cells. In addition, the NSCLC patients with high Id1 expression in primary tumor tissues had a better survival rate than the patients with low Id1 expression after surgical resection followed by the definitive chemotherapy. These findings suggest that high Id1 expression could enable the cancer cells to rely on Id1 for survival more than other surviving pathways as a result of oncogene addiction [20-22], which may hence provide a novel insight for evaluation of the prognostic role and therapeutic potential of Id1 in NSCLC.

## RESULTS

### Effect of paclitaxel and cisplatin on Id1 protein expression and cytotoxicity in NSCLC cells

**Figure 1:**
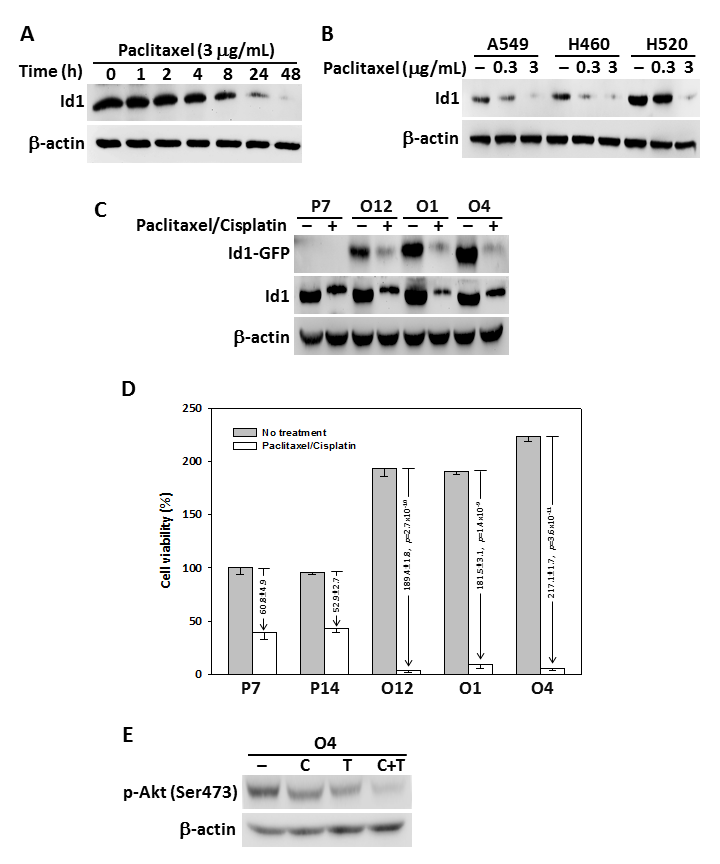
Paclitaxel and cisplatin suppressed Id1 expression and cell viability in NSCLC cells (A) Paclitaxel suppressed Id1 expression in H520 cells in a time-dependent manner. (B) Paclitaxel suppressed Id1 expression in A549, H460, and H520 cells in a dose-dependent manner. (C) Decreased Id1 expression in vector control H460 cells (P7) and Id1-overexpressing H460 cells (O1, O4 and O12) upon treatment with paclitaxel and cisplatin. (−: no treatment, +: co-treatment with paclitaxel and cisplatin). (D) Cell viability at 48 h after co-treatment with paclitaxel and cisplatin was suppressed to a larger degree in Id1-overexpressing H460 cells (O1, O4 and O12) compared with the vector control H460 cells (P7 and P14). *P* was analyzed by two-sided Student's *t*-test. (E) Expression of p-Akt (Ser473) in Id1-overexpressing H460 cells (O4) when treated with paclitaxel and/or cisplatin.

To study the effect of paclitaxel on Id1 protein expression in NSCLC cells, H520 cells were treated with paclitaxel and a time-dependent decrease in Id1 expression was observed (Figure 1A). In addition, paclitaxel treatment of A549, H460, and H520 lung cancer cells suppressed Id1 expression in a dose-dependent manner (Figure 1B). We then analyzed the inhibitory effect of co-treatment of paclitaxel and cisplatin on Id1 expression in Id1-GFP-overexpressing H460 cells. The results showed that Id1 expression was suppressed more in the Id1-GFP-overexpressing cells (clones O1, O4, and O12) than the vector control (clone P7) by the co-treatment (Figure 1C, middle panel). Intriguingly, while Id1-GFP overexpression (clones O1, O4, and O12) promoted cell proliferation in H460 cells, the cell viability was suppressed to a larger extent by co-treatment of paclitaxel and cisplatin in the Id1-GFP-overexpressing cells compared with the vector control (Figure 1D). As Id1 was known to promote Akt activation via phosphorylation in lung cancer and other caner types [15, 27, 28], we also examined whether treatment of paclitaxel and cisplatin affected the expression level of phosphorylated Akt. Our data revealed that the Akt phosphorylation (p-Akt Ser473) in Id1-overexpressing H460 cells (clone O4) was suppressed after treatment with paclitaxel or cisplatin, in particular when the two drugs were applied in combination (Figure 1E), implying that the effect of paclitaxel and cisplatin was mediated through Id1/Akt pathway.

**Figure 2:**
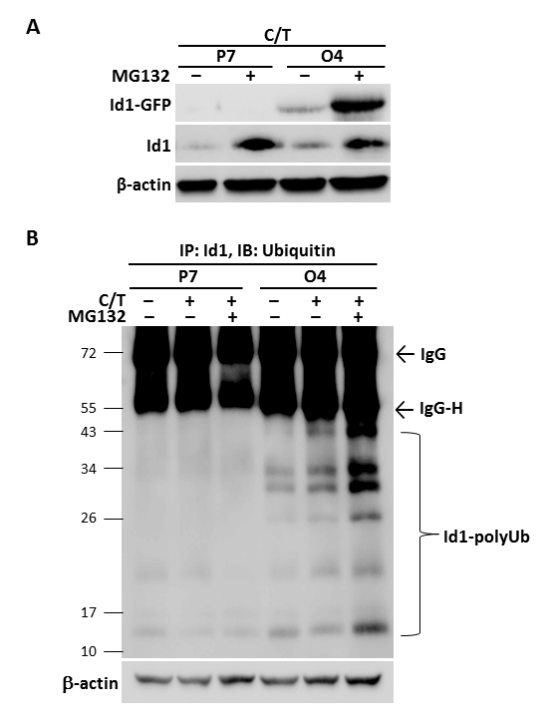
Paclitaxel and cisplatin down-regulated Id1 expression in NSCLC cells through protein ubiquitination/proteosome degradation system (A) Vector control H460 cells (P7) and Id1-GFP-overexpressing H460 cells (O4) were pre-incubated with or without MG132 (20 μM) for 1 h followed by co-treatment with paclitaxel and cisplatin for 24 h. (B) Vector control H460 cells (P7) and Id1-GFP-overexpressing H460 cells (O4) were pre-incubated with or without MG132 (20 μM) for 1 h, followed by co-treatment with paclitaxel and cisplatin for 8 h. Id1 ubiquitination was determined by immunoprecipitation with anti-Id1 antibody followed by immunoblotting for ubiquitin. β-actin served as an internal control.

It was reported that ubiquitination-proteasome system, one of the major protein degradation mechanisms [29], was related to the decrease of cellular Id1 [30]. Therefore, we sought to test whether the protein ubiquitination-proteosome was involved in the paclitaxel- and cisplatin-reduced Id1 expression. As shown in Figure 2A, the suppression of endogenous Id1 and overexpressed Id1-GFP in the Id1-GFP-overexpressing H460 cells (clone O4) upon treatment with paclitaxel and cisplatin was reversed when co-treated with the proteasome inhibitor MG132. In addition, the increased levels of ubiquitinated Id1 (Id1-polyUb) were detected in Id1-GFP-overexpressing H460 cells (clone O4) in the presence of MG132 by immunoprecipitation using anti-Id1 antibody and immunoblotting with anti-ubiquitin antibody (Figure 2B). Together, the results suggest that paclitaxel- and cisplatin-induced Id1 protein degradation was mediated through the ubiquitination-proteosome system.

### Effect of paclitaxel and cisplatin on xenograft NSCLC tumor growth in mice

**Figure 3:**
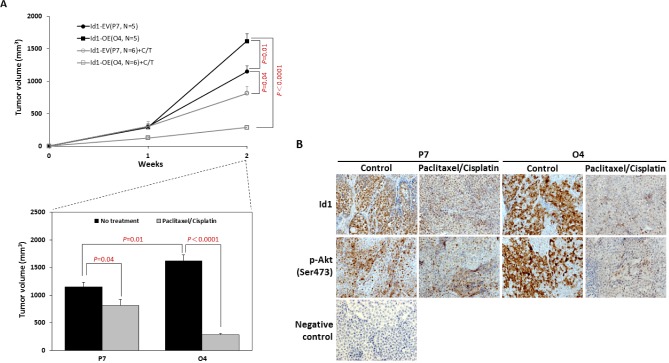
Co-treatment with paclitaxel and cisplatin led to a reduced tumor growth of NSCLC in mice xenograft model (A) (*Upper graph*) Time course of Id1-GFP-overexpressing (O4) and vector control (P7) xenograft tumor volume with or without treatment of paclitaxel and cisplatin. (*Lower graph*) Effect of paclitaxel and cisplatin treatment on Id1-GFP-overexpressing (O4) and vector control (P7) xenograft tumor volumes at the end of the xenograft assay. (B) Immunohistochemical staining for the expression of Id1 and p-Akt (Ser473) in xenograft tumors with or without paclitaxel and cisplatin treatment. Original magnification: 200×.

To further confirm the role of Id1 overexpression in lung tumor growth when treated with paclitaxel and cisplatin, the nude mice xenograft assay was employed. As shown in Figure 3A, tumor growth in the untreated Id1-GFP-overexpressing group (Id1-OE, O4) occurred at a faster rate than that in the untreated vector control group (Id1-EV, P7). However, there was a more dramatic reduction of the tumor observed when co-treated with paclitaxel and cisplatin in the Id1-GFP-overexpressing group (Id1-OE+C/T, O4) (*P* < 0.0001) compared with the vector control (Id1-EV+C/T, P7) (*P* = 0.04). The body weights of all mice were not significantly different when sacrificed after two weeks of the treatment (data not shown). Immunohistochemical analysis also revealed that the expression of Id1 and p-Akt (Ser473) was decreased upon co-treatment with paclitaxel and cisplatin, especially in the Id1-GFP-overexpressing group (Figure 3B).

### Association between Id1 expression levels in NSCLC tissue and patient survival after adjuvant chemotherapy with paclitaxel and cisplatin

**Figure 4:**
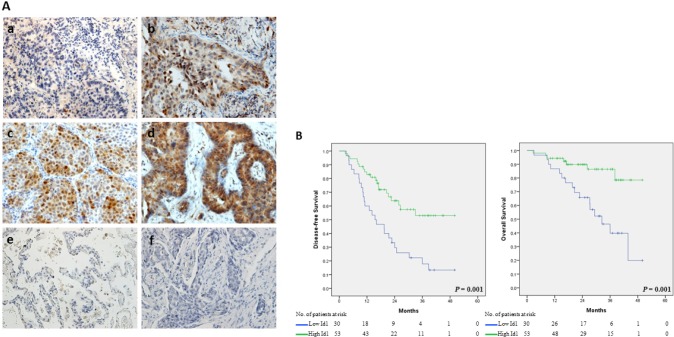
NSCLC patients with high Id1 expression showed improved disease-free survival and overall survival after adjuvant paclitaxel and cisplatin therapy (A) Id1 expression in lung cancer tissue (a-d) and in normal lung tissue (e) by immunohistochemistry. a-d: score 1-4 (defined in Materials and Methods); f: negative control. Original magnification: 200×. (B) Kaplan-Meier curve analysis of Id1 expression levels in association with disease-free and overall survival.

The expression of Id1 in lung cancer tissues was analyzed by immunohistochemistry, which was then correlated with the clinicopathological characteristics of surgically resected NSCLC patients with definitive adjuvant paclitaxel and cisplatin chemotherapy. Id1 protein expression levels were classified into four quartiles, with representative photographs shown in Figure 4A. Scores 1, 2 and 3 were categorized as low expression (36.1%), and score 4 was categorized as high expression (63.9%). The Id1 staining was predominantly observed in lung cancer tissues but not in the adjacent normal lung tissues (Figure 4A). An increased level of Id1 expression in lung cancer tissues was positively correlated with the increased tumor size (4.54±0.24 cm of the tumor in the high Id1 expression group *vs.* 3.70±0.24 cm of the tumor in the low Id1 expression group; *P* = 0.022) (Table 1). Notably, after receiving the adjuvant chemotherapy the patients with high Id1 expression in tumor tissues showed a better disease-free survival and overall survival than those with low Id1 expression by Kaplan-Meier survival analysis (Figure 4B). The mean of disease-free survival in the groups of high Id1 and low Id1 expression were 34.45±2.67 and 20.73±2.74 months, respectively. In addition, the mean of overall survival in the groups of high Id1 and low Id1 expression were 44.27±2.00 and 31.58±2.95 months, respectively.

**Table 1:** The average tumor size of NSCLC patients with low and high Id1 expression in primary cancer tissues

Variables	Patient No.	Low Id1 (n)		High Id1 (n)		*P* value *
Tumor size (cm)	83	3.70±0.24 (30)		4.54±0.24 (53)		0.022

* *P* value was calculated by Student's *t*-test.

Values were expressed as mean ± SEM.

To further investigate the association of Id1 expression and clinical parameters with disease-free survival, univariate and multivariable Cox regression analysis were applied. In the univariate analysis, statistically significant associations with disease-free survival were observed for the two parameters: lymph node status (*P* = 0.002 for N1 status and *P* = 0.001 for N2 status) and Id1 expression (*P* = 0.001) (Table 2). In the multivariate analyses, lymph node status (*P* = 0.015 for N1 status and *P* = 0.004 for N2 status) and Id1 expression (*P* = 0.024) also showed a statistical significance associating with the disease-free survival (Table 2).

**Table 2:** Univariate and multivariable analysis of disease-free survival for surgically treated NSCLC patients after adjuvant chemotherapy

Variables	Item	Univariate			Multivariable*		
		Hazard Rate Ratio	95% Confidence Interval	*P* value	Hazard Rate Ratio	95% Confidence Iinterval	*P* value
Age (y)	>70	1.46	(0.78, 2.72)	0.240	-	-	-
	≤70	1.00			-		
							
Sex	Male	0.93	(0.47, 1.83)	0.822	-	-	-
	Female	1.00			-		
							
Grade	III	1.38	(0.39, 4.89)	0.620	-	-	-
	II	1.82	(0.77, 4.34)	0.174	-	-	-
	I	1.00			-		
							
T status‡	T3	0.54	(0.19, 1.54)	0.249	-	-	-
	T2	0.76	(0.36, 1.60)	0.466	-	-	-
	T1	1.00			-		
							
N status‡	N2	3.60	(1.74, 7.43)	0.001	2.97	(1.42, 6.25)	0.004
	N1	3.43	(1.59, 7.41)	0.002	2.69	(1.21, 5.98)	0.015
	N0	1.00			1.00		
							
Histology	Squamous cell carcinoma	1.26	(0.67, 2.37)	0.478	-	-	-
	Adenocarcinoma	1.00			-		
							
Performance	1	1.66	(0.87, 3.17)	0.125	-	-	-
	0	1.00			-		
							
Smoking status	Current	0.85	(0.42, 1.71)	0.640	-	-	-
	Former	0.97	(0.47, 2.01)	0.939	-	-	-
	Never	1.00			-		
							
Id1	High	0.37	(0.20, 0.66)	0.001	0.49	(0.27, 0.91)	0.024
	Low	1.00			1.00		

* Variables with *p*<0.1 were included in the multivariable analysis.

‡ T, N statuses were based on the AJCC-TNM staging system [23].

We then analyzed the association of Id1 expression and clinical parameters with overall survival by univariate and multivariable Cox regression analysis. In the univariate analyses, statistically significant associations with overall survival were obtained for the following parameters: lymph node status (*P* = 0.006 for N1 status and *P* = 0.009 for N2 status), histology (*P* = 0.031), performance status (*P* = 0.005), and Id1 expression (*P* = 0.002) (Table 3). In the multivariate analysis, statistically significant associations with overall survival included lymph node status (*P* = 0.005 for N1 status and *P* = 0.009 for N2 status), histology (*P* = 0.003), performance status (*P* = 0.002), and Id1 expression (*P* = 0.022) (Table 3).

**Table 3:** Univariate and multivariable analysis of overall survival for surgically treated lung cancer patients after adjuvant chemotherapy

Variables	Item	Univariate			Multivariable*		
		Hazard Rate Ratio	95% Confidence Iinterval	*P* value	Hazard Rate Ratio	95% Confidence Interval	*P* value
Age (y)	>70	1.63	(0.68, 3.91)	0.278	-	-	-
	≤70	1.00			-		
							
Sex	Male	1.20	(0.45, 3.24)	0.718	-	-	-
	Female	1.00			-		
							
Grade	III	3.35	(0.56, 20.10)	0.186	-	-	-
	II	2.89	(0.67, 12.52)	0.157	-	-	-
	I	1.00			-		
							
T status‡	T3	0.85	(0.23, 3.22)	0.815	-	-	-
	T2	0.66	(0.22, 2.02)	0.468	-	-	-
	T1	1.00			-	-	-
							
N status‡	N2	4.27	(1.43, 12.75)	0.009	4.88	(1.48, 16.11)	0.009
	N1	4.90	(1.56, 15.33)	0.006	5.78	(1.68, 19.83)	0.005
	N0	1.00			1.00		
							
Histology	Squamous cell carcinoma	2.49	(1.09, 5.70)	0.031	4.35	(1.67, 11.33)	0.003
	Adenocarcinoma	1.00			1.00		
							
Performance	1	3.25	(1.42, 7.41)	0.005	4.59	(1.77, 11.92)	0.002
	0	1.00			1.00		
							
Smoking status	Current	1.98	(0.76, 5.17)	0.164	-	-	-
	Former	1.55	(0.54, 4.49)	0.419	-	-	-
	Never	1.00			-		
							
Id1	High	0.25	(0.10, 0.61)	0.002	0.34	(0.14, 0.86)	0.022
	Low	1.00			1.00		

* Variables with *p*<0.1 were included in multivariable analysis.

‡ T, N statuses were based on the AJCC-TNM staging system [23].

## DISCUSSION

In our clinical analysis, the size of primary tumor of NSCLC patients with high Id1 expression was in average larger than that of the patients with low Id1 expression before the definitive adjuvant chemotherapy (Table 1), implying that Id1 acted as a negative prognostic factor in NSCLC. In spite of the primary tumor size, the NSCLC patients with high Id1 expression showed a better survival rate than the patients with low Id1 expression after the adjuvant chemotherapy (Figure 4), suggesting that Id1 predicts a favorable prognosis under the specified treatment condition. It might seem paradoxical at first, however, the different prognostic role of Id1 could be reasoned by the occurrence of oncogene addiction.

Oncogene addiction is a phenomenon regarding the dependence of cancer cells on a particular oncogene for survival over other surviving pathways [20-22]. For example, it was reported that imatinib, an inhibitor of Bcr-Abl, selectively induced cytotoxicity in the Bcr-Abl-transfected HL-60 leukemic cells compared with the Bcr-Abl-negative parental cells [31]. On the other hand, the Bcr-Abl mutant (T315I)-transfected K562 leukemic cells showed resistance to the killing by imatinib in comparison to the Bcr-Abl-positive parental cells, suggesting that these cancer cells relied on Bcr-Abl for survival to a large degree (i.e. addiction to Bcr-Abl) [32, 33]. In another study, it was found that there was a reciprocal regulation between B-Myb and MYCN in neuroblastoma, and the tumor cells were addicted to B-Myb in a MYCN-dependent manner for cell proliferation [34]. Furthermore, treatment of c-Myc-expressing myeloma cells with 10058-F4, a specific inhibitor of c-Myc/MAX heterodimerization, resulted in an increase of cytotoxicity, suggesting that there was an addiction to c-Myc for survival in these cancer cells [35, 36]. There are also different types of oncogene addiction in relation to cellular activities. For example, the Ras-mediated tumor growth was dependent on the elevated levels of autophagy (i.e. autophagy addiction) [37]. More recently, Hall *et al*. reported that melanoma cells were addicted to glycolytic activity, which was up-regulated by the presence of mutant B-Raf (V600E) in these cancer cells [38]. Our current data of *in vitro* and *in vivo* model assays indicated that, while overexpression of Id1 promoted the cancer growth of NSCLC cells, co-treatment of paclitaxel and cisplatin caused a greater effect of reduction on cancer cell growth in the Id1-overexpressing NSCLC cells compared with the control cells (Figure 1D and 3). Along with our clinical analysis (Figure 4), these results suggest that NSCLC cells expressing high levels of Id1 may show a preference to rely on Id1 for survival (i.e. addiction to Id1), and therefore targeting Id1 in NSCLC cells with high Id1 expression would be considered a potentially effective therapeutic strategy.

It has been reported that adjuvant chemotherapies are advantageous for disease-free and overall survival among the patients with surgically resected NSCLC [2, 3, 39, 40]. Several biomarkers were reported as predictors for NSCLC patients receiving adjuvant chemotherapies, such as βIII tubulin, ERCC1, p27, p53, Ras, and Id1 [18, 41-43]. While these clinical studies showed the application of biomarkers for prediction of treatment outcome, the molecular mechanisms underlying the clinical responses remain largely unknown. In this study, we demonstrated for the first time that the decreased level of Id1 was caused by paclitaxel and cisplatin treatment (Figure 1C), and the reduction of Id1 under this treatment was mediated through a mechanism involving ubiquitination/proteasome degradation system (Figure 2). Besides, treatment of Id1-overexpressing NSCLC cells with paclitaxel and cisplatin led to a reduced level of phosphorylated Akt at Ser473 (Figure 1E), which was known a downstream signaling molecule of Id1 in NSCLC and other cancer cells [15, 44]. Since there are different signaling molecules (e.g. MAPK, PKC, mTOR, ATM, etc.) involved in Akt activation via Ser473 phosphorylation [45-48], it would be worthy to further investigate which of these molecules plays a role in the Id1/Akt pathway of the paclitaxel- and cisplatin-treated NSCLC.

It was noticed that the prognostic role of Id1 in our clinical analysis for survival in the NSCLC patients underwent adjuvant chemotherapy (Figure 4) appeared to be different from the findings of Ponz-Sarvisé *et al*. [18], whose report showed that Id1 was a poor prognostic factor in NSCLC patients. The reason for this discrepancy is unclear. However, it may result from the dissimilar clinical settings inherited in these analyses. For example, a single treatment modality (i.e. adjuvant chemotherapy) was employed in our current study, whereas there was more than one treatment modality (i.e. adjuvant chemotherapy, or radiotherapy combining adjuvant chemotherapy) given to the patients in their report [18]. As there have been a variety of adjuvant chemotherapies developed for NSCLC [49, 50], it will be valuable to determine whether Id1 also plays a role in NSCLC patients treated with different regimens of adjuvant chemotherapies.

In conclusion, our data suggest that Id1, a generally negative prognostic factor, served as a favorable prognostic factor in the case of surgically resected NSCLC patients receiving adjuvant paclitaxel and cisplatin chemotherapy. The differential roles of Id1 may be reasoned as a result of Id1 addiction in NSCLC cells for survival in the presence of paclitaxel and cisplatin treatment. Together, our current study provides a novel view of Id1 in the prognosis and therapeutic potential for NSCLC patients.

## MATERIALS AND METHODS

### Chemicals

Chemotherapeutic agents used in this study included paclitaxel (Taxol^®^, Bristol-Myers Squibb, USA) and cisplatin (Platinex^®^, Bristol-Myers Squibb, Italy). RPMI-1640 medium were obtained from Invitrogen (Carlsbad, CA). Fetal bovine serum (FBS) was obtained from Hyclone (Logan, UT). Penicillin, streptomycin, and amphotericin B were obtained from Biological Industries (Haemek, Israel). Other chemicals were obtained from Sigma (St. Louis, MO). Antibodies recognizing p-Akt1 (Ser473) (rabbit polyclonal, 1:1000), ubiquitin (rabbit monoclonal, 1:1000), and β-actin (rabbit monoclonal, 1:1000) were purchased from GeneTex (Irvin, CA). Anti-Id1 antibody (rabbit polyclonal, 1:200) was purchased from Santa Cruz Biotechnology (Santa Cruz, CA).

### Patient samples

Lung cancer tissues and adjacent non-cancer tissues were obtained from patients receiving surgical treatment at E-DA Hospital, Taiwan, from February 2007 to February 2013. This study was approved by the Institutional Review Board of E-DA Hospital, and informed consents were obtained from all patients. Staging was classified according to the American Joint Committee on Cancer (AJCC) tumor-node-metastasis (TNM) staging system [23]. Eighty-three surgically treated NSCLC patients with six cycles of adjuvant chemotherapy for four weeks post-operative were included in this study. The adjuvant chemotherapy regimen consisted of paclitaxel administered at a dose of 80 mg/m^2^ body surface area on days 1, 8, and 15, and cisplatin administered at a dose of 60 mg/m^2^ body surface area on day 15. The resected specimens were processed for tissue microarray and immunohistochemical staining.

### Tissue microarray

Tumor tissues and paired normal lung tissues for tissue microarray were obtained from formalin-fixed and paraffin-embedded tissue blocks. The construction of the tissue microarray was performed using an Alphelys BoostArrayer device (Plaisir, France) as described in a previous report [24]. Following staining with hematoxylin and eosin, the sections of microarray for tumor and normal tissues were examined by a pathologist for classification.

### Immunohistochemical analysis

Immunohistochemical staining and image processing were performed as described previously [24, 25]. In brief, after staining for Id1 the images were captured by a Nikon Eclipse 80i microscope (Tokyo, Japan) and processed using Adobe Photoshop 8.0 software. The positive Id1 immunostaining in the cytoplasm was determined and scored by two independent experts under the same examining condition. The results were divided into four quartiles: score 1, ≤ 25% positive cells; score 2, 26%–50% positive cells; score 3, 51%–75% positive cells; and score 4, ≥ 76% positive cells. Scores 1, 2 and 3 were categorized as low expression, and score 4 was categorized as high expression for statistical analysis.

### Cell culture

Three NSCLC cell lines (A549 lung epithelial carcinoma, H460 lung large cell carcinoma, and H520 lung squamous cell carcinoma) were obtained from the Bioresource Collection and Research Center (BCRC) (Hsinchu, Taiwan). The genotypes and phenotypes of the cell lines were authenticated by the BCRC. Cells were cultured in RPMI-1640 medium supplemented with 10% FBS and antibiotics. All cells were grown at 37°C in a 5% CO_2_ incubator.

### Stable transfection for Id1 overexpression

Full length cDNA for Id1 was cloned into the pINCY vector backbone for fusion with the green fluorescent protein (GFP) tag (Id1-GFP). H460 cells were seeded on a 6-cm dish (Corning Life Sciences, Corning, NY) at a density of 5.6×10^5^ cells per dish and incubated overnight at 37°C in a 5% CO_2_ atmosphere. For each transfection, 26.2 μL of lipofectamine LTX (Invitrogen, Carlsbad, CA) was added to 1413.5 μL of Opti-MEM (Invitrogen) and incubated for 5 min at room temperature. The diluted plasmid (7.1 μg) containing either Id1-GFP or empty vector as control was mixed with lipofectamine solution and incubated for 30 min at room temperature before added to cells. The transfected H460 cells were then grown in RPMI-1640 media with 1 μg/ml puromycin for stable clone selection.

### XTT colorimetric assay

Cell viability was determined by the method of tetrazolium salt XTT assay (Roche Life Science, Indianapolis, IN). After treatment for 48 h with paclitaxel and cisplatin, the cell culture media were removed and XTT assay was carried out as described in a previous article [25]. Three independent experiments with six replicates in each were performed.

### Immunoblotting analysis

Immunoblotting analysis for protein expression was carried out by Western blot as described previously [26], and the chemiluminescent signal was captured by a UVP BioSpectrum 500 imaging system (Upland, CA). Three independent experiments were performed.

### Immunoprecipitation

To study the ubiquitination of Id1, immunoprecipitation was performed by the Catch and Release v2.0 Reversible Immunoprecipitation System (Millipore, Billerica, MA) according to the manufacturer's instruction. Briefly, the harvested cells were washed with ice-cold phosphate buffered saline (PBS) and resuspended in solubilization buffer (25 mM Tris pH 7.6, 150 mM NaCl, 1% Nonidet P-40, 5% glycerol, 1 mM PMSF, 1 mM NaF, 1 mM Na_3_VO_4_, and 1 μg/mL aprotinin). Insoluble materials were removed by centrifugation (13,000 g, 10 min at 4°C), and the soluble cell lysates (1.5 mg) were incubated with 4 μg of anti-Id1 antibody at 4°C overnight. The Catch and Release column, which contained a proprietary resin with high affinity for binding to the antigen-antibody complex, were used to pull down the protein samples. The column was then washed with salt-free wash buffer (1% Nonidet P-40, 0.25% deoxycholic acid, and 15 mM imidazole), followed by centrifugation (2,000 g, 1 min at room temperature). Immunoprecipitated proteins were eluted from the resin by incubation with an elution buffer provided by the manufacturer for 30 min at 37°C, followed by immunoblotting analysis. An aliquot of the cell lysates (50 μg) was served as input control. Three independent experiments were performed.

### Nude mice xenograft study

All experiments involving mice were performed according to the guidelines of the Animal Committee and approved by the Institutional Review Boards of E-DA Hospital and I-Shou University. Four-week-old female immunodeficient (Foxnlnu/Foxnlnu) mice, obtained from the National Science Council Animal Center of Taiwan, were used in this study with the procedures reported in a previous article [15]. Briefly, when the subcutaneously implanted tumors became visible (approximately 50 mm^3^ in size), the mice were randomly assigned to be treated intraperitoneally with PBS or paclitaxel (3 μg/g body weight) plus cisplatin (5 μg/g body weight) every other day for two weeks. Tumor sizes were measured by calipers every other day and their volumes were calculated according to the formula of (width^2^ × length)/2.

### Statistical analysis

All statistical analyses were performed using the SPSS v19 statistical package for PC (IBM Corp., Armonk, NY). Comparisons of the average tumor size between the high and low Id1 expression group were analyzed by two-sided Student's *t*-test. Survival curves were generated using Kaplan-Meier estimates, and the significance of differences between curves was evaluated by the log-rank test. Furthermore, hazard ratios (HRs) and 95% confidence intervals (CIs) computed from univariate and multivariable Cox regression models were used for investigating the relationship between clinicopathological characteristics and survival. Statistical significance was accepted at *P* < 0.05.
